# Confinement of Dirac fermions in gapped graphene

**DOI:** 10.1038/s41598-024-61539-9

**Published:** 2024-06-24

**Authors:** Fatemeh Pakdel, Mohammad Ali Maleki

**Affiliations:** Department of Physics, University of Zanjan, University Blvd., Zanjan, 45371-38791 Iran

**Keywords:** Electronic properties and materials, Electronic properties and devices, Electronic properties and devices, Two-dimensional materials

## Abstract

We explore the electronic transport characteristics of gapped graphene subjected to a perpendicular magnetic field and scalar potential barriers. Employing the Dirac–Weyl Hamiltonian and the transfer-matrix method, we calculate the transmission and conductance of the system. Our investigation delves into the impact of the energy, the gap energy parameter ($\Delta$) and the magnetic flux parameters, including the number of magnetic barriers (*N*), the magnetic field strength (*B*) and the width of the magnetic barriers. We demonstrate that manipulating energy and total magnetic flux parameters allow precise control over the range of incident angle variation. Moreover, adjusting the tunable parameter $\Delta$ effectively confines quasiparticles within the magnetic system under study. Notably, an increase in N results in a strong wave vector filtering effect. The resonance effects and the peaks in the transmission and conductance versus $\Delta$ are observed for $N>1$. The tunability of the system’s transport properties, capable of being toggled on or off, is demonstrated by adjusting $\Delta$ and *B*. As $\Delta$ or *B* increases, we observe suppression of the transmission and conductance beyond critical parameter values.

## Introduction

In recent years, graphene has gained significant attention due to its intriguing physical properties, particularly chiral and massless electron excitations, which hold promise for various applications and scientific studies^1–4^. However, certain phenomena, such as Klein tunneling through electrostatic barriers in graphene, may impose limitations on its potential use in nanoelectronic devices^5^. To harness graphene’s potential, most applications necessitate a means of controlling the transport of charge carriers, known as Dirac–Weyl (DW) quasiparticles^6^, which can be achieved through the application of an external magnetic field^7–10^. Upon the application of a critical low magnetic field, a collapse transition affects the Landau levels, leading to the classical manifestation of the Lorentz force^11,12^. Simultaneous application of magnetic fields and electrostatic potentials gives rise to additional intriguing phenomena including giant magnetoresistance modulation^13^, the Fano resonance^14^, resonance splitting^15^, Fabry-Pérot interference^16^ and the collapse of Landau levels^12^.

A natural approach to confine charge carriers and suppress Klein tunneling is to open a band gap near the Dirac points^17,18^. Various methods can be employed to generate this gap in graphene^19^, such as finite size effects achieved by shaping graphene into nanoribbons^20,21^. In this context, the electron constriction within the ribbon depends on its edges and width^22,23^. Gap engineering can also be achieved through spin-orbit interaction^24^, the interaction of suitable elements and adsorption^25–29^, doping the graphene with chemical species^30^ and placing graphene on top of an appropriate substrate which induces an intrinsic Dirac mass for charge carriers^31^. Another approach involves covering monolayer and bilayer graphene with water and ammonia molecules, inducing a gap in the spectrum^32^. An experimental study has shown that a tunable gap can be generated by doping monolayer graphene grown on a SiC (0001) substrate with low-energy (5eV) Li+ ions^33^. Similarly, a significant adjustable gap of up to 740 meV can be created by adsorption of Na onto bare and Ir cluster superlattice-precovered epitaxial graphene on Ir(111)^34^. In another research, the impact of gap fluctuation on the transmission and conductance of monolayer and bilayer graphene superlattices, characterized by a series of scalar potential barriers, has been investigated^35^. In both cases, gap fluctuations lead to the suppression of Klein tunneling and a reduction in system conductance. Also, a theoretical research has demonstrated the possibility of highly valley-polarized electron transport in a gapped graphene film with a magnetic-electric barrier structure, which arises from a valley-dependent phase mechanism^5^. Another study has concluded that a specific value of the gap induces fermion confinement and suppresses Klein tunneling and conductance in gapped graphene with a delta-function-like magnetic barrier^36^. Confining electrons in transmission through one barrier is conducted better than two barriers. The investigation of magnetic confinement of quasiparticles^3^ and the wave vector filtering effect^37^ in purely magnetic systems has been extensively researched. Similarly, studies have explored systems featuring pure electrostatic barriers^38^. However, these studies predominantly focus on gapless graphene. It is imperative to extend these investigations to encompass gapped graphene systems. Specifically, our aim is to identify parameters governing the confinement of quasiparticles in gapped graphene. Our particular interest lies in scrutinizing the influence of the gap parameter on the transport characteristics of the system consisting of magnetic barriers in conjunction with electrostatic barriers.

The primary objective of this study is to investigate the influence of the gap parameter on a graphene sheet with a finite number of both magnetic barriers (square-shaped) and electrostatic potential barriers. The gap energy parameter, denoted by $\Delta$, acts as a switch controlling the transport properties of the system. Tuning $\Delta$ together with adjusting the energy, and the total magnetic flux parameters including the number of magnetic barriers *N*, strength of the magnetic field *B*, and width of the magnetic barriers can modulate the transmission and the conductance. The wave vector filtering effect is observed as *N* increases and also in the vicinity of energies close to the height of the scalar potential barrier. Increasing the total magnetic flux parameters or reducing the energy, induces angular confinement of transmission. For critical values of $\Delta$ and *B*, transmission and conductance are suppressed. While the conductance decreases continuously with increasing *B* and mainly $\Delta$, a few numbers of peaks in transmission and conductance are observed for $N>1$. As *N* increases in the magnetic structure under our study, the forbidden zone of transmission and the conductance becomes wider and the oscillations are created in it.

The structure of this manuscript is organized as follows: In “Model and theoretical method”, we introduce the model Hamiltonian for a gapped graphene sheet in presence of magnetic field and electrostatic scalar potential and employ the transfer matrix method and Landauer–Büttiker formalisms to calculate the transmission and total conductance of the system. The physical findings are presented in “Results and discussion” to obtain the dependence of transmission and conductance on various parameters introducing the system and “Conclusion” summarizes the conclusions drawn from this research.

**Figure 1:**
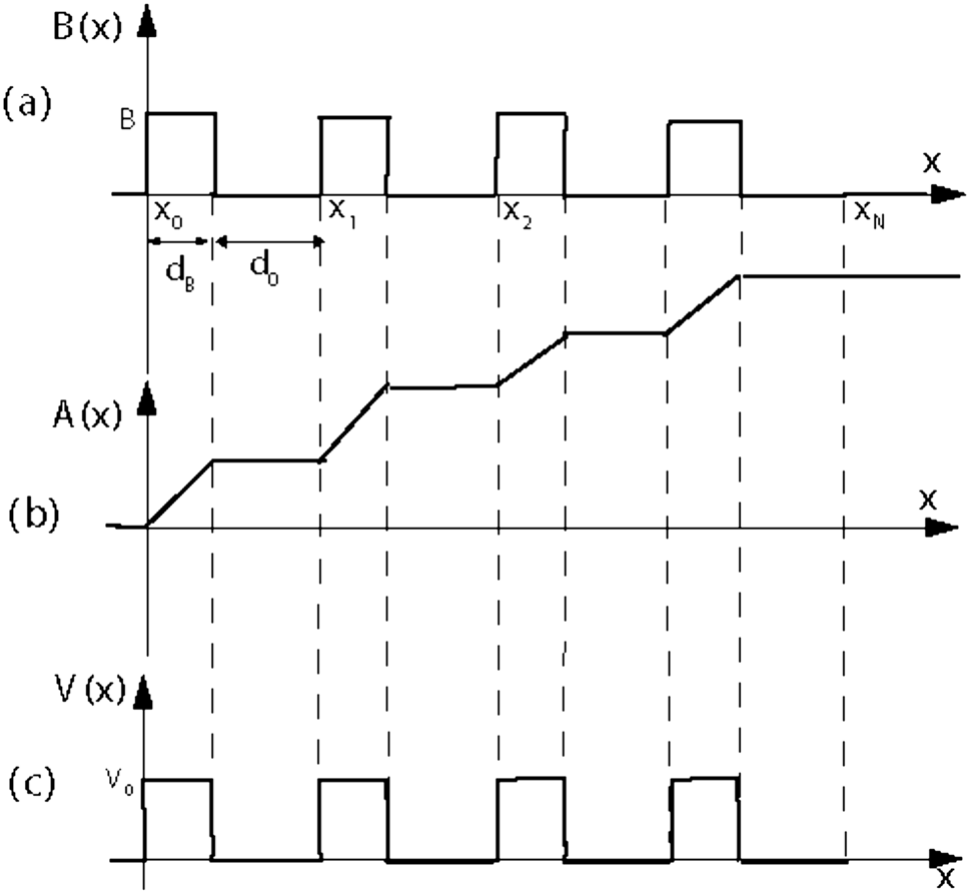
(**a**) The magnetic profile consisting of *N* magnetic barriers of width $d_B$ and height *B* separated by magnetic wells of width $d_{0}$ with each other. (**b**) The vector potential profile A(x). (c) The scalar potential barrier profile with width $d_B$ and height $V_0$.

## Model and theoretical method

We consider a monolayer graphene sheet positioned in the *x*–*y* plane, featuring a gap in its energy spectrum. This sheet is subjected to a perpendicular magnetic field while being influenced by a scalar potential. The magnetic field, denoted as $\varvec{B}=B(x)\varvec{e}_z$, exhibits an alternative spatial variation, as depicted in Fig. 1a. This field is associated with a vector potential $\varvec{A}=A(x)\varvec{e}_y$, where $B_z=\partial _xA(x)$, as illustrated in Fig. 1b. The profile of this magnetic field encompasses a series of *N* magnetic barriers, each characterized by a height *B* and a width $d_B$. Additionally, there exists a scalar potential with a magnitude of $V_0$ within the magnetic regions defined as:

1 $$\begin{aligned} V(x)=\left\{ \begin{array}{ll} V_0, &{} x_n\le x<x_n+d_B; \\ 0, &{} \hbox {otherwise.} \end{array} \right. \end{aligned}$$

This potential distribution is depicted in Fig. 1c, where $x_n=n(d_B+d_0)$, $n=0, \ldots , N-1$ and $d_0$ represents the width of the non-magnetic regions. The magnitude of the vector potential, as shown in Fig. 1b, can be expressed as:

2 $$\begin{aligned} A(x) =\left\{ \begin{array}{ll} 0, &{} x\le 0; \\ (nd_B+x-x_n)B, &{} x_n\le x<x_n+d_B; \\ (n+1)Bd_B, &{}x_n+d_B\le x<x_{n+1}; \\ NBd_B, &{} x\ge x_N. \end{array} \right. \end{aligned}$$

Here, the quantity $NBd_B$ serves as the total magnetic flux, introduced as a key parameter controlling transmission and conductance^6^.

The model described above can be realized if the monolayer graphene covered by a thin dielectric layer. The uniform band gap is created by the sublattice-dependent graphene–substrate interaction. A ferromagnetic stripe which is deposited on the dielectric layer can act as a magnetic barrier and, also, applying voltage to the ferromagnetic stripe can induce the electric barrier^5^. We employ the two-band Dirac–Weyl Hamiltonian to describe our system^1^, expressed as:

3 $$\begin{aligned} H(x)=v_{\text {F}}\varvec{\sigma }.(-i\hslash \varvec{\nabla }+e\varvec{A}(x))+V(x)\mathbb {I}_2+\Delta \sigma _z. \end{aligned}$$

Here $\varvec{\sigma }=(\sigma _x,\sigma _y)$ and $\sigma _z$ represent the Pauli matrices and $v_{\text {F}}$ ($\sim c/300$) corresponds to the Fermi velocity. The inclusion of the energy gap, $\Delta$, in the Hamiltonian, Eq. (3), causes electrons to exhibit characteristics akin to massive Dirac fermions^35^. Notably, the y-component of momentum, $k_y$, remains conserved, allowing us to express the entire wave function as:

4 $$\begin{aligned} \Psi (x,y)=e^{ik_yy} \begin{pmatrix} \psi _1(x)\\ \psi _2(x) \end{pmatrix}. \end{aligned}$$

In order to express the eigenvalue equation for the Hamiltonian given by Eq. (3) in a dimensionless form, we employ the typical magnetic field magnitude $B_0$ and the magnetic length scale $l_0=\sqrt{\hslash /(eB_0)}$. This results in the following dimensionless form of the eigenvalue equation:

5 $$\begin{aligned} \begin{pmatrix} V(x)+\Delta -E &{} -i\partial _x-i[k_y+A(x)] \\ -i\partial _x+i[k_y+A(x)] &{} V(x)-\Delta -E \end{pmatrix} \Psi (x,y)=0. \end{aligned}$$

Here, we have scaled all relevant quantities such as $k_y$, *A*(*x*), length scales, and energy scales to units of $l_0^{-1}$, $l_0B_0$, $l_0$ and $\hslash v_{\text {F}}/l_0$, respectively. With $B_0=0.1$ T as our reference magnetic field scale, we find that the length scale $l_0$ is 81.13 nm, and the energy scale $\hslash v_{\text {F}}/l_0$ is 8.113 meV. Within the magnetic regions, when employing Eqs. (4) and (5), we obtain a pair of coupled differential equations for the spinors $\psi _1(x)$ and $\psi _2(x)$:

6a $$\begin{aligned} -i[\frac{d}{dx}+(k_y+A(x))]\psi _2(x)= & {} [E-V_0-\Delta ]\psi _1(x), \end{aligned}$$

6b $$\begin{aligned} -i[\frac{d}{dx}-(k_y+A(x))]\psi _1(x)= & {} [E-V_0+\Delta ]\psi _2(x). \end{aligned}$$

Eliminating $\psi _2(x)$ between Eqs. (6a) and (6b) yields:

7 $$\begin{aligned} \frac{d^2\psi _1(x)}{dx^2}-[(k_y+A(x))^2+\frac{dA(x)}{dx}-(E-V_0)^2+\Delta ^2]\psi _1(x)=0. \end{aligned}$$

We can introduce a new variable, $q=\sqrt{2/B}[k_y+A(x)]$, and a parameter, $p=[(E-V_0)^2-\Delta ^2]/(2B)- 1$. This allows us to rewrite Eq. (7) as:

8 $$\begin{aligned} \frac{d^2\psi _1(q)}{dq^2} - \left( \frac{q^2}{4} - p - \frac{1}{2}\right) \psi _1(q)=0, \end{aligned}$$

which is known as the Parabolic Cylinder equation^39^. It has two independent solutions, denoted as $D_{p}(q)$ and $D_{p}(-q)$. Consequently, the spinors of the full wave function in the magnetic regions can be expressed as:

9a $$\begin{aligned} \psi _1(x)= & {} cD_{p}(q)+dD_{p}(-q), \end{aligned}$$

9b $$\begin{aligned} \psi _2(x)= & {} \frac{i\sqrt{2B}}{E-V_0+\Delta }[cD_{p+1}(q)-dD_{p+1}(-q)], \end{aligned}$$

where *c* and *d* are constants. In obtaining Eq. (9b), we have used the recursion relation $(d/dz)D_p(z)-(1/2)zD_p(z)+D_{p+1}(z)=0$ for the parabolic cylinder functions^39^. For the non-magnetic regions, the wave function is obtained by solving Eq. (5), yielding:

10 $$\begin{aligned} \Psi _0(x,y)=e^{ik_yy} \left[ ae^{ik_xx} \begin{pmatrix} 1\\ (k_x+i[k_y+A(x)])/E' \end{pmatrix} +be^{-ik_xx} \begin{pmatrix} 1\\ (-k_x+i[k_y+A(x)])/E' \end{pmatrix} \right] , \end{aligned}$$

where *a* and *b* are constants, $E'=\sqrt{E^2-\Delta ^2}$, and $k_x=\sqrt{E'^2-[k_y+A(x)]^2}$.

The boundary conditions can be imposed at the boundaries $x=0, d_B, d_B+d_0, \ldots , x_{N-1}+d_B$ as follows:

11 $$\begin{aligned} \Psi _0(x_n,y)=\Psi _B(x_n,y),\,\,\,\Psi _B(x_n+d_B,y)=\Psi _0(x_n+d_B,y), \end{aligned}$$

where *n* runs from 0 to $N-1$, and $\Psi _B(x)$ represents the wave function within the magnetic regions. For simplicity, we will momentarily disregard the *y*-component and express the wave functions as:

12a $$\begin{aligned} \Psi _0(x)= & {} W_0(x) \begin{pmatrix} a\\ b \end{pmatrix}, \end{aligned}$$

12b $$\begin{aligned} \Psi _B(x)= & {} W_B(x) \begin{pmatrix} a\\ b \end{pmatrix}, \end{aligned}$$

where the matrices $W_0(x)$ and $W_B(x)$ are defined as:

13a $$\begin{aligned} W_0(x)= & {} \begin{pmatrix} e^{ik_xx} &{} e^{-ik_xx}\\ e^{ik_xx}(k_x+i[k_y+A(x)])/E' &{} e^{-ik_xx}(-k_x+i[k_y+A(x)])/E' \end{pmatrix}, \end{aligned}$$

13b $$\begin{aligned} W_B(x)= & {} \begin{pmatrix} D_{p}(q) &{} D_{p}(-q)\\ \frac{i\sqrt{2B}}{E-V_0+\Delta } D_{p+1}(q) &{} \frac{-i\sqrt{2B}}{E-V_0+\Delta } D_{p+1}(-q) \end{pmatrix}. \end{aligned}$$

By employing the boundary conditions expressed in Eq. (11), we can derive a transfer matrix *T* defined as:

14 $$\begin{aligned} \begin{pmatrix} a_0\\ b_0 \end{pmatrix} =T \begin{pmatrix} a_N\\ b_N \end{pmatrix}. \end{aligned}$$

Here, $a_0=1$ (representing the incoming electron from the left side), and $b_0=r$ (the reflection amplitude) correspond to the wave function in the region $x\le 0$. Similarly, $a_N=t\sqrt{k_x^i/k_x^f}$ (the transmission amplitude) and $b_N=0$ correspond to $x\ge x_{N-1}+d_B$. Here, $k_x^i$ ($k_x^f$) represents the *x*-component of the wave vector in the regions $x<0$ ($x>x_N$). The transfer matrix is calculated as:

15 $$\begin{aligned} T=W_0^{-1}(0)\,W_{B}(0)\,W_B^{-1}(d_B)\,W_{0}(d_B)\cdots W_{B}^{-1}(x_{N-1}+d_B)W_0(x_{N-1}+d_B). \end{aligned}$$

The transmission probability^6^ is expressed as:

16 $$\begin{aligned} \tau (E,k_y) = \left| t\right| ^2 = \frac{k_x^f}{k_x^i} \frac{1}{{\left| T_{11}\right| ^2}}. \end{aligned}$$

Here, $T_{11}$ represents the 11-element of the transfer matrix *T*. To calculate the zero-temperature conductance, we utilize the Landauer-Buttiker formalism^40^:

17 $$\begin{aligned} G(E) = G_0 \int _{-\pi /2}^{\pi /2} \tau (E,k_y) \cos {\varphi } d\varphi , \end{aligned}$$

where $G_0$ is defined as $2e^2EL_y/(\pi h)$, with $L_y$ representing the length of the graphene sample along the *y*-direction.

Finally, in the last part of this section, we draw attention to certain constraints related to the angles of incidence and the energies of incident electrons. We parameterize the momenta in the initial and final regions using the incidence angle ($\varphi$) and emergence angle ($\varphi _f$) as follows: $k_y=E'\sin \varphi$ for the initial region and $k_y+NBd_B=E'\sin \varphi _f$ for the final region. The conservation of $k_y$ uniquely determines the emergence angle via $\sin \varphi +NBd_B/E'=\sin \varphi _f$, resulting in the condition:

18 $$\begin{aligned} \left| \sin \varphi +NBd_B/E'\right| \le 1. \end{aligned}$$

This condition signifies that, for all incident angles, the transmission is entirely suppressed when $\left| E'\right| \le NBd_B/2$. It is worth noting that all the upcoming figures are available in color online.

**Figure 2:**
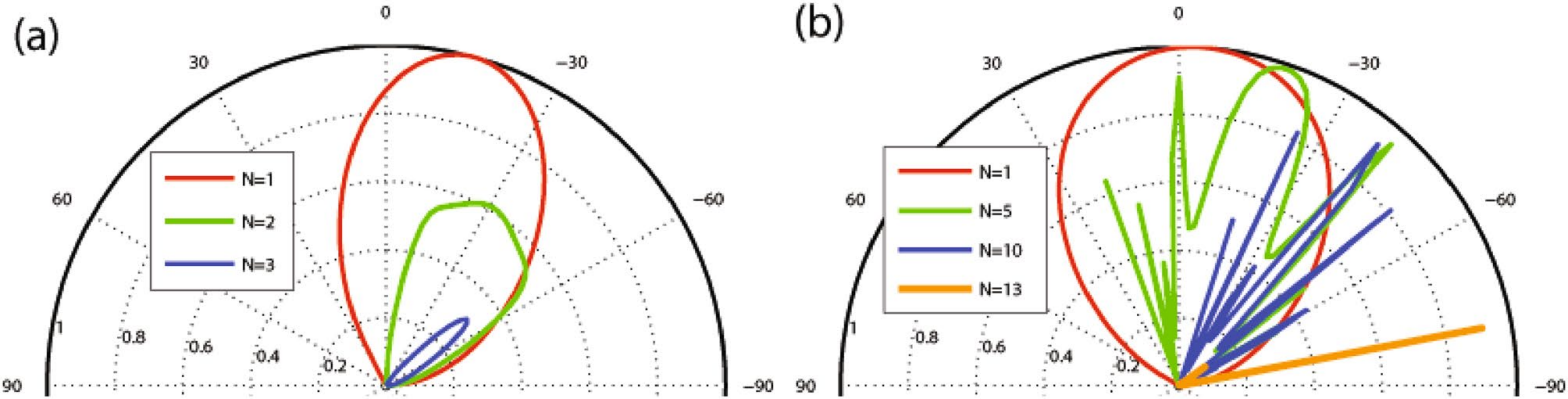
Angular dependence of the transmission for $E=2$, $V_0=7$, $\Delta =0.5$ and different values of *N* with (**a**) $B=1$, $d_B=1$ and (**b**) $B=0.5$, $d_B=0.5$.

**Figure 3:**
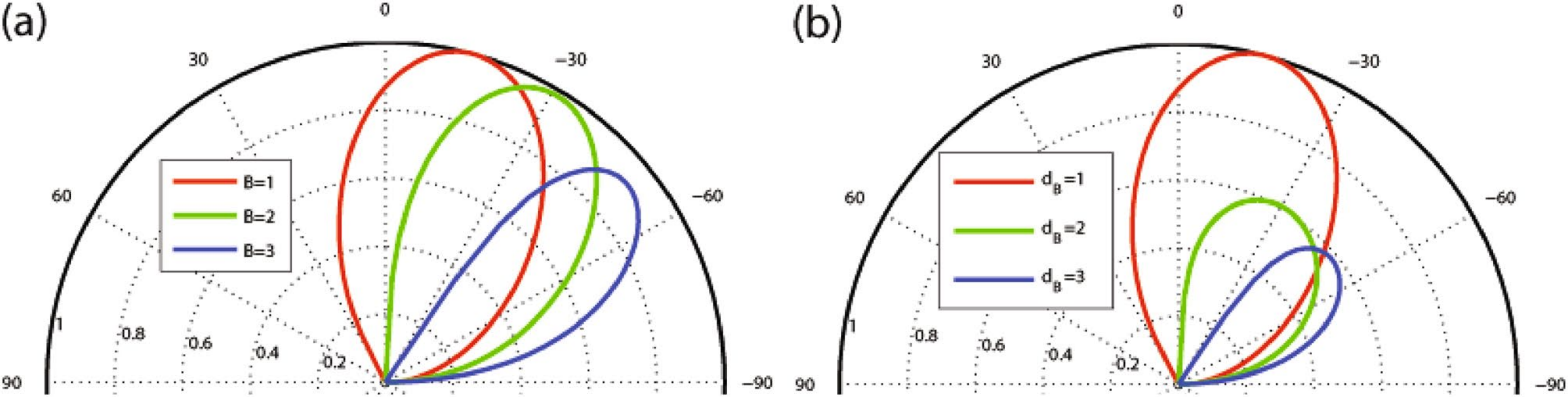
Angular dependence of the transmission for $N=1$ (**a**) varying *B* and (**b**) varying $d_B$. Other parameters are the same as in Fig. 2a.

## Results and discussion

In this section, we present the results of our numerical analysis for the transmission and conductance, computed using Eqs. (16) and (17). Our focus is on analyzing the effects of various parameters, including the energy gap, incident angle, energy, strength of the magnetic field, widths, and the number of magnetic barriers, on the behavior of transmission and conductance through the structure depicted in Fig. 1. We examine the phenomenon of strong wave vector filtering and transmission suppression within the constraints imposed by Eq. (18). Throughout all figures, we consider $d_0=10$. Before proceeding, it’s essential to note that, as per Eq. (18), the incident angle $\varphi$ must satisfy the condition:

19 $$\begin{aligned} \varphi \le \varphi _c, \end{aligned}$$

where $\varphi _c=\sin ^{-1}\left( 1-NBd_B/E'\right)$ represents the maximum allowable incident angle. For $\varphi >\varphi _c$, there is a forbidden zone with no transmission. Additionally, in the case of normal incidence, the energy and the energy gap must satisfy the following constraints:

20 $$\begin{aligned} \left| E\right| \ge \sqrt{\Delta ^2+\left( \frac{NBd_B}{1-\sin \varphi }\right) ^2},\,\,\,\,\, \left| \Delta \right| \le \sqrt{E^2-\left( \frac{NBd_B}{1-\sin \varphi }\right) ^2}. \end{aligned}$$

These conditions show the forbidden zone of transmission and conductance and are applicable to all the figures that follow.

**Figure 4:**
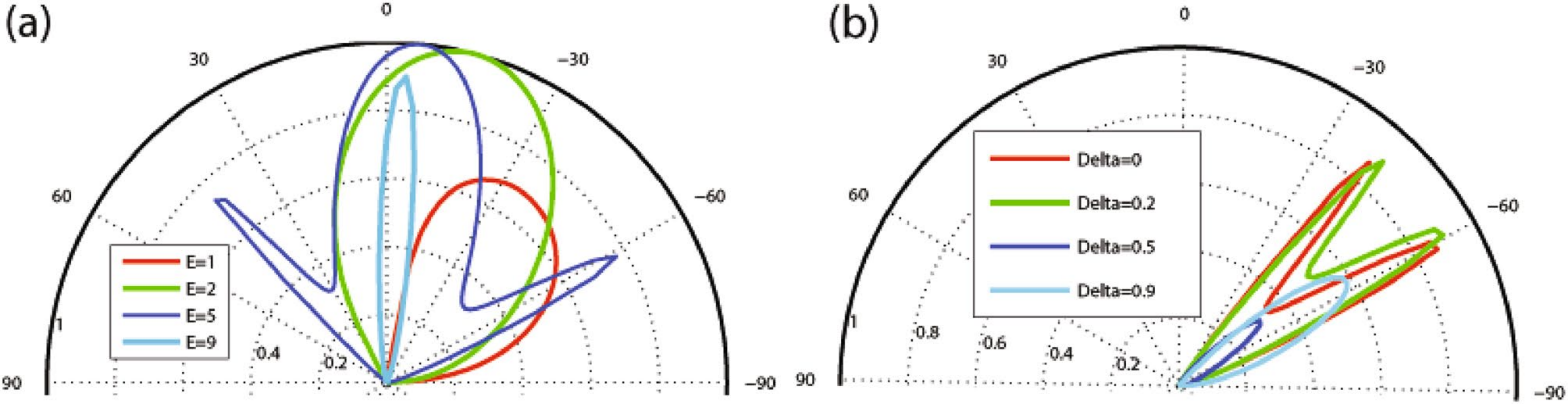
Angular dependence of the transmission for (**a**) $N=1$, $V_0=10$ and different values of *E* and (**b**) $N=3$, $V_0=7$ and different values of $\Delta$. Other parameters are the same as in Fig. 2a.

We commence our inquiry and numerical analyses by examining the angular dependence of the transmission in our studied magnetic system. In Fig. 2, we present the dependence of transmission probability on the incident angle for different values of the number of magnetic blocks, *N*. The parameters used here are $E=2$, $V_0=7$, $\Delta =0.5$ with (a) $B=1$, $d_B=1$ and (b) $B=0.5$, $d_B=0.5$. The transmission lobes become narrower and shorter while they shift towards negative angles as *N* increases. The range of variation in the incident angle decreases and the wave vector filtering effect is observed by increasing *N*. In this scenario, the quasiparticles are permitted to transmit exclusively within defined and restricted incident angles. This effect holds practical potential as an angular switch for confining DW quasiparticles within systems incorporating magnetic and electric barriers. In Fig. 2a the maximum angles $\varphi _c$ for $N=1, 2$ and 3 are $\varphi _c=28.9^\circ , -1.9^\circ$ and $-33.3^\circ$, respectively, in agreement with relation (19) while there is not any allowed value for $\varphi$ in the case of $N>3$. In Fig. 2b, due to the new values of *B* and $d_B$, we are able to increase the number of magnetic blocks up to $N=13$. For enough large values of *N*, some oscillations are created in the angular profile. Notably, the Klein tunneling effect is not observed due to the absence of perfect normal transmission (Fig. 2a), but it is observed for small values of the total magnetic flux $NBd_B$, in the case of $N=1$ (Fig. 2b). Figure 3 illustrates the transmission as a function of the incident angle for the case of a single magnetic block ($N=1$), varying the magnetic field strength (Fig. 3a) and the width of the magnetic blocks (Fig. 3b). The lobes become narrower and tend towards negative angles as *B* or $d_B$ increase. Both the transmission and $\varphi _c$ decrease with increasing *B* and $d_B$, consistent with relation (19). We observe that the magnetic parameters *B* and $d_B$ are effective factors to control the transmission and its angular range which can lead to the confinement of the charge carriers in the gapped graphene. In Fig. 4, the angular dependencies of transmission are shown for different values of energy (Fig. 4a) and $\Delta$ (Fig. 4b). We have chosen $V_0=10$ in Fig. 4a, in order to be able to examine the behavior of the transmission for higher energies. The transmission of the system is not, mainly, affected by changing $\Delta$ in the single barrier case, so we examine the dependence for $N=3$, shown in Fig. 4b. The critical angle $\varphi _c$ increases with an increase in *E* or a decrease in $\Delta$ consistent with the relation (19). When *E* approaches $V_0$, known as the equal-barrier case, the transmission decreases, and the lobes shrink, leading to the wave vector filtering effect where the quasiparticles with a small angular range are transmitted. Perfect transmission is observed for certain energy values, and the lobes split into three branches around $E=V_0/2$. Figure 4b shows that the magnetic system with three blocks exhibits a wave vector filtering, for $\Delta =0.5$, due to the small range for the incident angle. In summary, Figures 2, 3 and 4 collectively demonstrate that increasing parameters related to the total magnetic flux (*N*, *B*, and $d_B$) and $\Delta$ or decreasing *E* results in a reduced range of incident angle variation. This effect manifests as angular filtering, which can be manipulated by adjusting these parameters.

**Figure 5:**
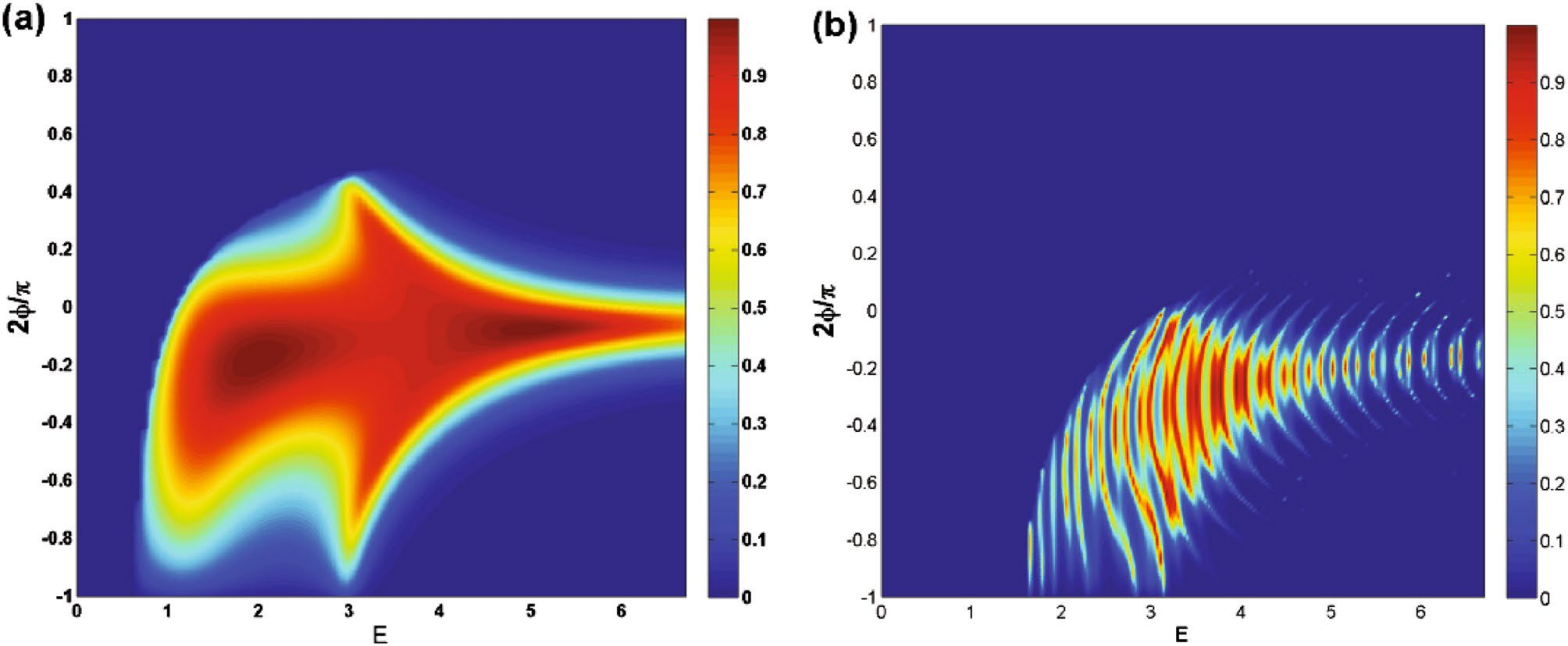
The transmission probability versus $\varphi$ and *E* for (**a**) $N=1$ and (**b**) $N=3$. Other parameters are the same as in Fig. 2a.

**Figure 6:**
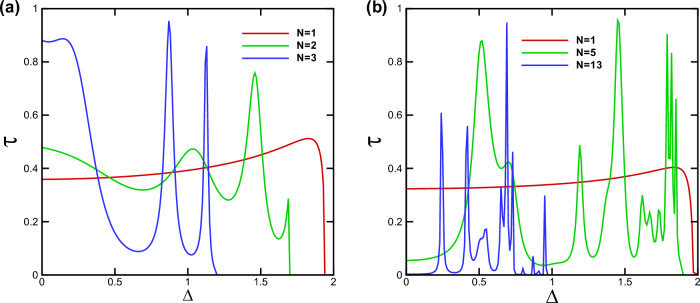
The transmission probability as a function of $\Delta$ for different values of *N* for $\varphi =-60^\circ$ with (**a**) $B=d_B=1$ and (**b**) $B=d_B=0.5$. Other parameters are the same as in Fig. 2a.

**Figure 7:**
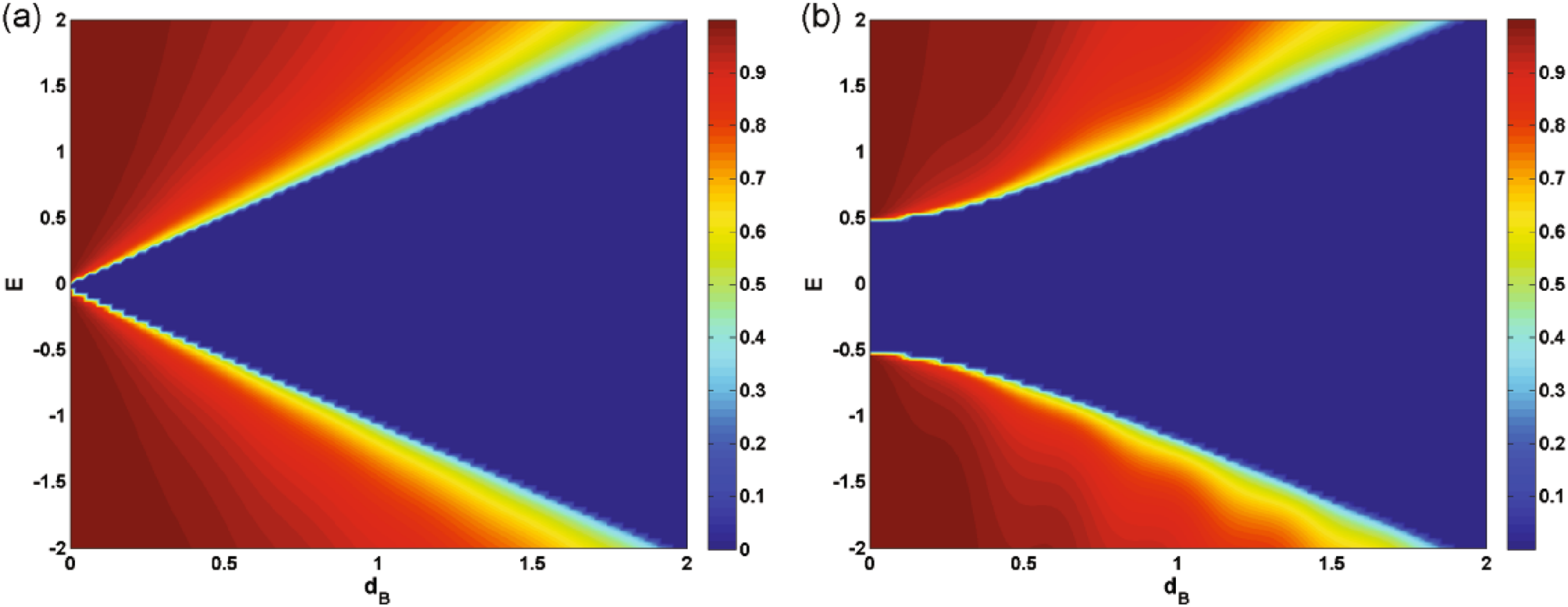
The normal transmission versus *E* and $d_B$ for $N=1$ with (**a**) $\Delta =0$ and (**b**) $\Delta =0.5$. Other parameters are the same as Fig. 2a.

**Figure 8:**
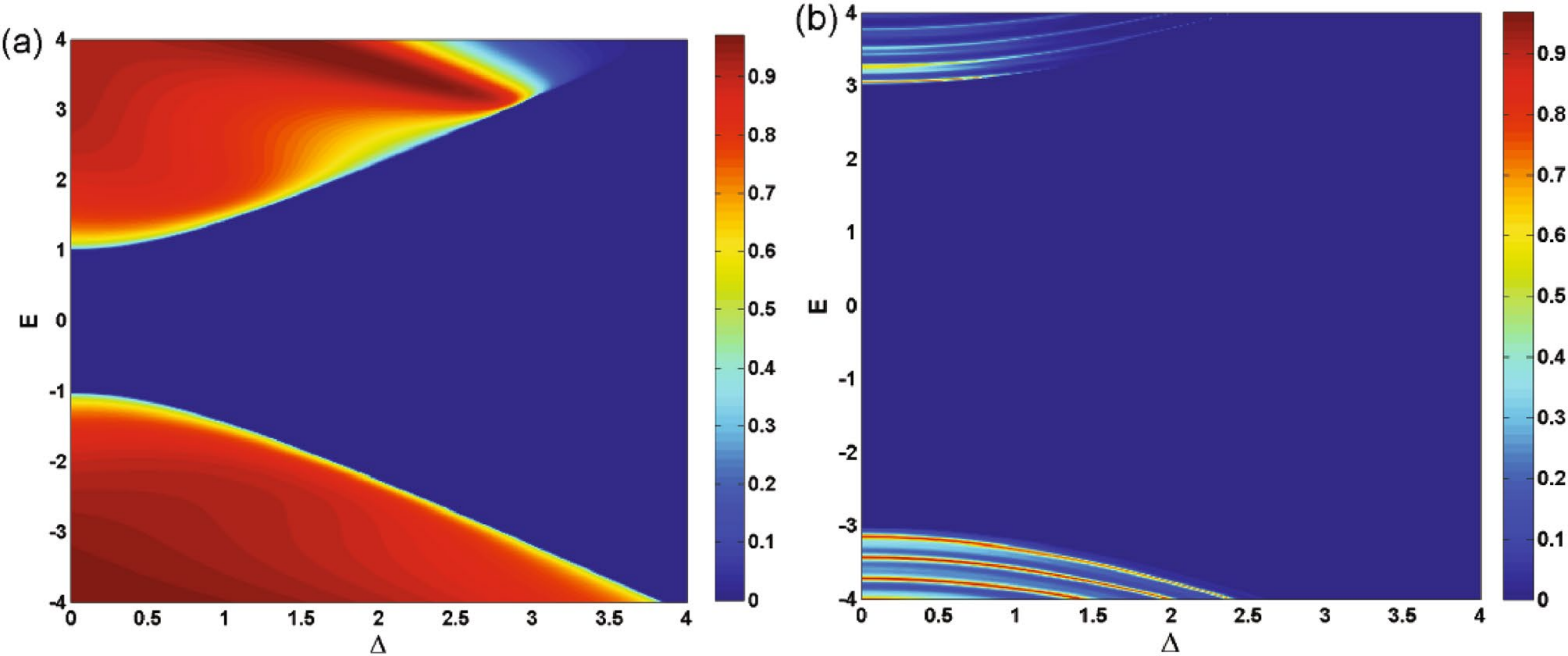
The normal transmission versus *E* and $\Delta$ for (**a**) $N=1$ and (**b**) $N=3$. Other parameters are the same as Fig. 2a.

In Fig. 5, we present the density plots of the transmission versus the incident angle and the energy for (a) $N=1$ and (b) $N=3$. We observe that increasing *N*, the area of the forbidden zone of transmission increases and the range of allowed angles decreases in the negative angles. Perfect transmission is observed close to the normal angles for special values of the energy and $N=1$ (Fig. 5a). A strong wave vector filtering effect is observed close to the equal-barrier energies in negative incident angles in the vicinity of the normal incidence, as shown in Fig. 5a. This effect happens in discrete energies, for $N>1$ (Fig. 5b). So, by choosing a single or several number of magnetic barriers together with a suitable scalar potential and appropriate incident energies can be utilized to design an efficient wave vector filter for charge carriers in gapped graphene. The oscillations in transmission and the resonance effect are observed for $N>1$. This suggests possible applications as magnetic switches for quasiparticles transport in the gapped graphene, in practice. In Fig. 6, we plot the transmission as a function of $\Delta$ for $\varphi =-60^\circ$ and various numbers of magnetic barriers with (a) $B=1$, $d_B=1$ and (b) $B=0.5$, $d_B=0.5$. It is evident that the transmission is suppressed beyond a critical value of $\Delta$ dictated by relation (20). This critical value decrease by increasing *N*. By assigning new values to the magnetic parameters *B* and $d_B$, in Fig. 6b, we arrive at larger values of *N* in the allowed zone where the fluctuations have increased compared to Fig. 6a. For cases with $N>1$, we observe oscillations and resonances in the transmission. Altering the gap parameter, $\Delta$, modulates the transmission behavior. The density plots illustrating the normal ($\varphi =0$) transmission are presented in Fig. 7, showcasing the dependence on both energy and the width of the magnetic barrier for the case of a single magnetic barrier. We consider two scenarios: one without the gap parameter (Fig. 7a) and the other with $\Delta =0.5$ (Fig. 7b). In Fig. 7a, the boundaries of the forbidden zone of transmission are defined by $E=\pm d_B$, as derived from relation (20). On the other hand, in Fig. 7b, the boundaries of the forbidden zone of transmission are given by $E=\pm \sqrt{d_B^2+\Delta ^2}$, reflecting the influence of the gap parameter. Increasing $d_B$ expands the width of the forbidden zone, consistent with relation (20). Figure 8 displays the normal transmission versus both energy and the gap parameter for cases with $N=1$ and $N=3$. As expected, increasing *N* and $\Delta$ leads to a wider range for the forbidden zone of transmission, as indicated by relation (20). The widening of the energy gap with increasing *N* has been reported previously for similar magnetic systems^37^. The parameters *N*, $\Delta$ and $d_B$ can control the boundaries of the forbidden zone of transmission and are important in switching electronic devices. For $N>1$, the normal transmission exhibits oscillatory behavior versus energy (Fig. 8b), which becomes more pronounced for oblique incident angles (see also Fig. 5b).

**Figure 9:**
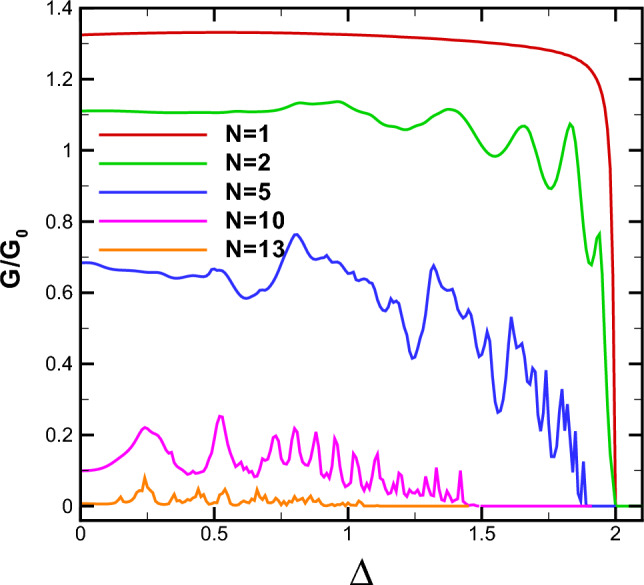
The conductance as a function of $\Delta$ for $B=d_B=0.5$ and different values of *N*. Other parameters are the same as in Fig. 2a.

**Figure 10:**
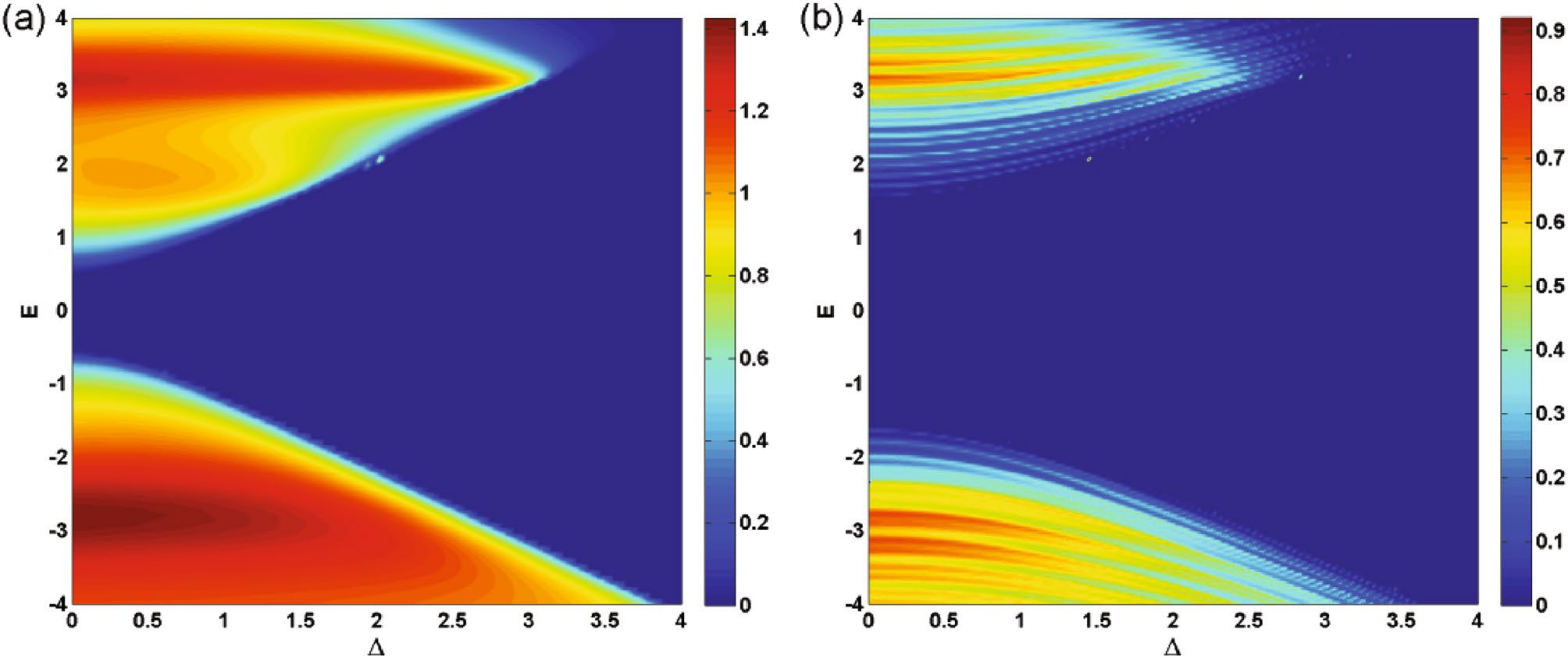
The conductance versus *E* and $\Delta$ for (**a**) $N=1$ and (**b**) $N=3$. Other parameters are the same as in Fig. 2a.

**Figure 11:**
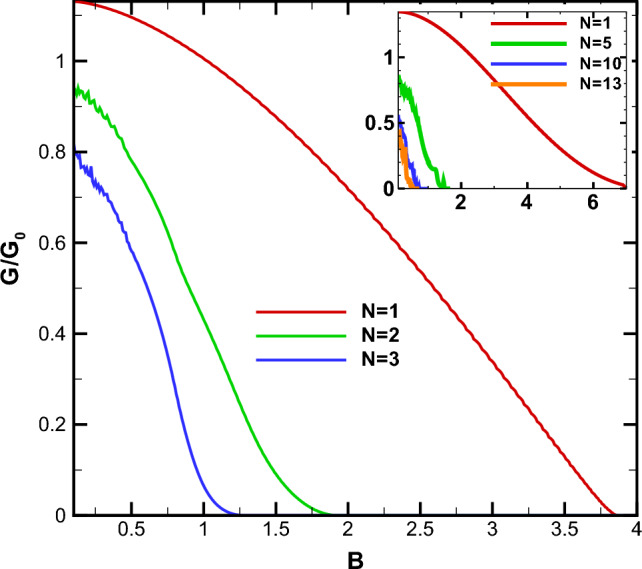
The conductance as a function of *B* for different values of *N*. Other parameters are the same as in Fig. 2a. The inset is sketched for $d_B=0.5$.

The conductance of the magnetic system is computed by averaging the transmission over all possible incident angles, as described in Eq. (17). Thus, all the discussions done, up to now, about the transmission is reflected in the system’s conductance. In Fig. 9, we present the conductance of the studied system as a function of the gap energy for $B=0.5$, $d_B=0.5$ and different numbers of magnetic barriers. The conductance decreases as the gap parameter $\Delta$ increases. This reduction is uniform for $N=1$ while we observe some peaks, for $N>1$, similar to behaviour of the transmission in Fig. 6. Eventually, the conductance vanishes around the same critical values of $\Delta$ as in Fig. 6b. This critical value decreases by increasing *N* and can be considered as an efficient parameter in controlling and confinement of Dirac fermions. The oscillations in the conductance for $N>1$ can be interpreted as confinement and control mechanisms for the transport properties of the DW quasiparticles. Additionally, the density plots for the conductance of the system are displayed in Fig. 10, illustrating the dependence on both energy and the gap parameter for cases with $N=1$ and $N=3$. The forbidden zone of conductance is also observed here, like the same as for the transmission in Fig. 8. The area of the forbidden zone increases with an increase in *N*. Although the range of the forbidden zone of the conductance is the same as for the normal transmission in one magnetic barrier case (Fig. 8a), it decreases for $N>1$ (Fig. 8b). This indicates that the oblique incident angles have a greater impact on reducing the range of the forbidden zone. The conductance exhibits oscillations for $N>1$, and it decreases with an increase in *N*, similar to what was in the case of the transmission. Finally in Fig. 11, we depict the conductance as a function of the magnetic field strength for various numbers of magnetic barriers. The conductance uniformly decreases with an increase in *B* and eventually vanishes at a critical value, $B_c$. The oscillations observed in the conductance for small values of *B* and $N>1$ in Fig. 11, which is also observed in Fig. 10b, were previously reported in an experimental work^41^. The inset is sketched for $d_B=0.5$ in order to be able to increase *N* up to $N=13$ in the allowed zone. Increasing *N* decreases $B_c$ and the oscillations increases. We conclude that the conductance of the system can be controlled by varying parameters such as *E*, *B*, $\Delta$ and *N*.

## Conclusion

We conducted an investigation into the electronic transport properties of a graphene sheet with an energy gap, under the influence of both magnetic and scalar potential barriers with specific configuration. Our study reveals that it is possible to confine and manipulate DW quasiparticles in the gapped graphene by tuning the magnetic flux, energy and the gap parameter. We observed a wave vector filtering effect as we increased the number of barriers; see Fig. 2. Additionally, we found that the range of variation in the incident angle narrows and stretches towards negative angles when the parameters of total magnetic flux (*N*, *B* and $d_B$) are increased or *E* is decreased, in accordance with relation (19). It should be noted that we observed Klein tunneling only for the case $N=1$ and small values of the total magnetic flux, $NBd_B$. Negative angles contributed more significantly in the conductance and reduced the forbidden zone of transmission and conductance for $N>1$. Our research demonstrates that the transmission decreases and lobes contract around $E=V_0$, indicating wave vector filtering effect. Moreover, we observed perfect transmission and lobe splitting for energies close to $V_0/2$ in the case of $N=1$. The gapped graphene shows perfect transmission for special values of energy and incident angle. By increasing *N*, the strong wave vector filtering and resonance effects are observed for a few discrete values of the energy and the oscillations in the transmission are created; see Fig. 5b. The area of the forbidden zone is increased, by increasing *N*.

Our findings highlight the strong correlation between the transport properties of the magnetic system and the gap parameter, $\Delta$. The measurable gap can be experimentally generated using various methods, as detailed in Refs.^33,34^. In practical devices, such as Bragg Reflectors, the Dirac fermions can be more effectively confined by simultaneously adjusting both *B* and $\Delta$, in conjunction with modifying the forms of the potential barriers^42^. We identified a critical value of $\Delta$ at which the system’s transmission and conductance are suppressed, in accordance to the relation (20). For specific values of $\Delta$, we observed resonance peaks, while some other values leads to deeps. Thus, the tunable gap energy can serve as an efficient parameter to control the transport properties of DW quasiparticles. Altering $\Delta$ also influences the boundaries of the forbidden zone of transmission and conductance. Increasing *N* widens the gap, as illustrated in Figs. 8 and 9. Moreover, the conductance of the system decreases as *N* increases, with the appearance of oscillations. Finally, we found that the conductance uniformly decreases with increasing *B*, and a critical value, $B_c$, exists at which conductance is significantly suppressed. The magnetic barriers, similar to what we studied, have been investigated extensively as fabricated on GaAs 2-dimensinal electron gas^43–45^. The phenomena explored in this study can serve as a valuable foundation for experimental works related to switchable devices and may have practical implications for the design of graphene-based electronic devices.

## Data Availability

The datasets used and/or analysed during the current study available from the corresponding author on reasonable request.
